# Effectiveness of a parent-focused intervention targeting 24-hour movement behaviours in preschool-aged children: a randomised controlled trial

**DOI:** 10.1186/s12966-024-01650-2

**Published:** 2024-09-09

**Authors:** Jie Feng, Wendy Yajun Huang, Cindy Hui-Ping Sit, John J. Reilly, Asaduzzaman Khan

**Affiliations:** 1 Academy of Wellness and Human Development, Hong Kong Baptist University, Hong Kong, China; 2 Department of Sports Science and Physical Education, The Chinese University of Hong Kong, Hong Kong, China; 3 Dr. Stephen Hui Research Centre for Physical Recreation and Wellness, Hong Kong Baptist University, Hong Kong, China; 4 Physical Activity and Health Group, School of Psychological Sciences and Health, University of Strathclyde, Glasgow, Scotland; 5 School of Health and Rehabilitation Sciences, The University of Queensland, Brisbane, Australia

**Keywords:** Physical activity, Sedentary behaviour, Sleep, Preschooler

## Abstract

**Background:**

Interventions focusing on individual behaviours (physical activity, sedentary behaviour, sleep) of preschool-aged children have been widely studied. However, there is a lack of understanding about integrated interventions that target all three 24-hour movement behaviours. This is the first study to assess the effectiveness of an intervention aimed at improving all three 24-hour movement behaviours among preschoolers in Hong Kong.

**Methods:**

A 12-week randomised controlled trial with a 12-week follow-up was conducted. Parent-child pairs were randomised to integrated approach (targeting all three behaviours), dyadic approach (targeting physical activity and sedentary behaviour including screen time), or wait-list control group. Utilising the Internet-based delivery, this intervention consisted of education materials, workshops, and interactive questionnaires and reminders. Two intervention groups employed the same strategies, with the only difference being that the integrated approach targeted sleep in addition to physical activity and sedentary behaviour. The outcomes were preschoolers’ overall 24-hour movement behaviours which were assessed by the Activity Sleep Index (ASI), movement behaviour composition, and absolute duration of movement behaviours. Generalised estimating equations were conducted to evaluate the intervention.

**Results:**

A total of 147 preschoolers (4.8 ± 0.9 years old, 56.5% boys) and their parents were included. Preschoolers in all groups had a lower ASI at follow-up compared with baseline. Preschoolers in the integrated approach had a smaller decline in ASI at follow-up, compared to that in the control group (3.41; 95% confidence interval [CI] = 0.07, 6.76). Preschoolers in both intervention groups had a smaller reduction of the composition of time spent in physical activity at follow-up, and a decreased screen time at postintervention and follow-up. No significant differences were found for the sleep subcomponent. Furthermore, preschoolers in the dyadic approach had a smaller increase in the sedentary behaviour subcomponent (vs. control: − 0.21; 95% CI = − 0.37, − 0.05) at follow-up.

**Conclusions:**

Both intervention groups showed a decrease in screen time at postintervention, but there were no significant changes in other behaviours. The favourable changes observed at follow-up demonstrated the effectiveness of both intervention approaches on alleviating the decline in the composition of time spent in physical activity and reducing screen time and revealed the possible effectiveness of the integrated approach in promoting overall movement behaviours among preschoolers.

**Trial registration:**

The study is prospectively registered at the Chinese Clinical Trial Registry (ChiCTR2200055958).

**Supplementary Information:**

The online version contains supplementary material available at 10.1186/s12966-024-01650-2.

## Introduction

Movement behaviours (physical activity, sedentary behaviour, sleep) developed in the early years are associated with numerous health outcomes and could impact their future life [1]. Individually, physical activity [2], sedentary behaviour [3], and sleep [4], are closely linked to various aspects of health during early childhood, including physical fitness, motor development, cognitive function, and cardiometabolic health. Over the past decade, there has been an increasing focus on the concept of the 24-hour day, with growing recognition that all movement behaviours should be considered in combination. A systematic review for the early years (0–4 years) recommended that, to achieve optimal health, the ideal combination is higher physical activity, lower sedentary behaviour, and sufficient sleep [1]. In light of this, the World Health Organisation launched the 24-hour movement guidelines for children under five years [5]. Specifically, in a 24-hour day, preschoolers are recommended to have at least 180 min of total physical activity including 60 min of moderate-to-vigorous intensity physical activity (MVPA); no more than 60 min of sedentary screen time; and 10–13 h of good quality sleep [5]. However, a systematic review including 8,943 children from 11 countries in the early years reported that only 13% of them met the recommended levels of all three guidelines [6]. In Hong Kong, a low prevalence (2.9%) of meeting all three guidelines was also observed among preschoolers aged between three and six years [7]. It is, therefore, imperative to develop interventions that effectively optimizing multiple time-use behaviours in the early years.

Interventions aiming at improving all three behaviours in a 24-hour day are still in their infancy. To the best of our knowledge, there have been limited interventions aiming at changing all three time-use behaviours [8, 9]. Specifically, one intervention among adolescents exhibited improvement in all behaviours after one academic year [8], while another intervention among school-aged children demonstrated a lack of favourable changes across nearly all behaviours [9]. Another parent-focused intervention aimed at improving the composition of 24-hour movement behaviours among toddlers is still ongoing, with no findings available yet [10]. No ‘integrated’ intervention, targeting all three movement behaviours, has been conducted among preschoolers. In contrast, single behaviour change interventions have been mainstream for a long time and their effectiveness on behaviour change has been well-documented in previous systematic reviews [11–13]. Specifically, for children aged 0 to 5 years old, a 3-minute increase in MVPA was observed in physical activity interventions [11]; a 19-minute decrease in sedentary behaviour was observed in interventions targeting sedentary behaviour [12]; sleep-focused interventions increased sleep duration by an average of 9 min per night [13]. In addition, previous multiple-behaviour interventions [14, 15] have primarily focused on daytime behaviours including physical activity and sedentary behaviour which are not independent of each other. However, given that the finite nature of time in a 24-hour day and the co-dependence of all behaviours, theoretically, changes in time spent in one behaviour means the reallocation of time in other behaviours [16]. Collectively, an integrated intervention targeting all three 24-hour movement behaviours simultaneously is warranted, providing a more feasible and flexible approach towards health promotion.

While behaviour change interventions for children under five years have primarily taken place in preschools and childcare settings, there is growing evidence that highlights the importance of parental engagement in facilitating behaviour improvement [16]. Following the aforementioned promise of single-behaviour interventions, it has been recommended to explore the effectiveness of integrated interventions encompassing all three movement behaviours in a family-based setting [16]. Additionally, school closures and increased parenting time resulting from the Coronavirus Disease 2019 (COVID-19) pandemic underscore the importance and timeliness of a parent-focused intervention. Therefore, this intervention was designed to (1) examine the effectiveness of a parent-focused 24-hour movement behaviour intervention among preschoolers; (2) explore if the integrated approach (physical activity + sedentary behaviour + sleep intervention) is more effective than dyadic approach (physical activity + sedentary behaviour intervention) in increasing physical activity, decreasing sedentary behaviour, and improving sleep; and (3) examine the feasibility and acceptability of both integrated and dyadic approaches. Being composed of three core components (attitude, subjective norm, perceived behavioural control), the Theory of Planned Behaviour was adopted as the theoretical framework in designing the intervention, incorporating behaviour change techniques [17], more details are described elsewhere [18]. We hypothesised that (1) both intervention groups would demonstrate effectiveness in changing movement behaviours favourably; (2) the integrated approach would yield greater effectiveness in improving movement behaviours, compared with the dyadic approach; and (3) both interventions would be feasible and acceptable.

## Materials and methods

This study adhered to the Consolidated Standards of Reporting Trials (CONSORT) Statement for randomised trials (http://www.consort-statement.org/). A detailed study protocol and main outcomes are described elsewhere [18]. Approval was obtained from the Research Ethics Committee, Hong Kong Baptist University (Ref. No.: SOSC-SPEH-2021-22-200) and written consent from parents was obtained.

### Participants

This three-arm randomised controlled trial was conducted in Hong Kong. Invitation letters were sent to all kindergartens in Hong Kong, and more families were invited using purposive and snowball sampling from May to July 2022, when schooling and regular work routines gradually resumed and social distancing measures were lifted after COVID-19 restrictions. This study recruited 165 parent-child pairs, of which 125 pairs were from eight kindergartens, and the remaining 40 pairs were recruited individually. After baseline screening using ActiGraph and questionnaires, 147 of them were included, including 111 parent-child pairs from eight kindergartens and 36 individually recruited pairs. Children who met all of the 24-hour movement guidelines (i.e., ≥ 180 min of total physical activity, including ≥ 60 min of MVPA, ≤ 1 h of sedentary screen time, and 10 to 13 h of sleep, in a day) [5] at baseline were excluded.

### Randomisation and blinding

A third party who was unaware of the purpose of this study performed randomisation using computer-generated random numbers. Following the collection of baseline data, parent-child pairs were assigned to one of three groups (physical activity + sedentary behaviour + sleep group [integrated approach], physical activity + sedentary behaviour group [dyadic approach], or wait-list control group) randomly, using a 1:1:1 allocation ratio.

### Sample size

Sample size calculation was performed using G*Power 3.1.9.7 with the “F test” family (ANOVA: Repeated measures, within-between interaction). Employing a three-arm, four-repeated-measure design, a total of 99 parent-child pairs are needed to detect an effect size *f* of 0.15 with a power of 0.90 (*α* = 0.05), assuming a correlation of 0.50 between repeated measures. A small effect size assumption was based on previous interventions targeting physical activity [11], sedentary behaviour [19], and sleep [20] among children aged 0 to 5 years old. Considering that less than 3% of preschoolers met all three guidelines at baseline [7] and taking into account a potential attrition rate of 20% [21], an over-sampling approach was employed to recruit 165 parent-child pairs, resulting in 147 pairs being deemed sufficient for the study.

### Intervention

The intervention was implemented in accordance with the protocol published previously [18]. In brief, families in the integrated approach attended a 12-week online intervention designed to help their preschool-aged children improve all three movement behaviours. The intervention incorporated several key components (Supplementary Table 1). Participants received biweekly educational materials via the WhatsApp, with each set of materials requiring approximately 20 min to review. The materials included individual reports on their children’s current behaviours, the gaps between their current behaviours and recommended guidelines, knowledge about the health benefits of improving behaviours, examples to improve behaviours, strategies for goal setting and habit development, and potential barriers and strategies. Additionally, three 30-minute workshops were delivered through Zoom meetings. Finally, participants completed biweekly interactive questionnaires using Google Forms, with each questionnaire taking roughly 10 min to finish. The dyadic approach employed the same intervention contents as the integrated approach, with the exception that the intervention materials were limited to physical activity and sedentary behaviour (including sedentary screen time). Families in the wait-list control group received no intervention. All groups attended four assessments at baseline, six weeks (mid-term), 12 weeks (postintervention), and 24 weeks (follow-up). The full details of interventions and procedures are described elsewhere [18].

### Primary outcomes

#### Activity sleep index

To summarise six dimensions of 24-hour movement behaviours (i.e., total physical activity, MVPA, screen time, sleep duration, sleep onset variability, and morning wake-time variability) among preschoolers, Activity Sleep Index (ASI) was created [22]. After rescaling each item from 0 to 10, a total score ranging from zero to 60 was obtained. A higher score indicates a more favourable overall pattern of movement behaviours. More details about the calculation is described elsewhere [18].

#### Compositional data

Isometric log-ratio coordinates (ilrs) were utilised to present the time spent on one behaviour compared to other behaviours, employing compositional data analysis. For example, the equation of compositional physical activity was as follows, presenting the time spent on physical activity relative to sedentary behaviour and sleep [23]:

$$\:ilr-physical\:activity=\sqrt{\frac{2}{3}}\text{ln}\left(\frac{PA}{\sqrt{SB*sleep}}\right)$$

Similarly, ilr-sedentary behaviour and ilr-sleep were obtained.

### Secondary outcomes (individual movement behaviours)

Physical activity, sedentary behaviour, and sleep were assessed by ActiGraph accelerometers (ActiGraph wGT3X-BT, Pensacola, Florida, USA), which were worn on children’s non-dominant wrists for 24-hours over seven consecutive days. Data were analysed using ActiLife software v6.13.4. The analysis included children who supplied data for at least one day with a minimum of 16 h of wearing time [24, 25]. Validated cut-off points for preschoolers were adopted. Specifically, sedentary time, light intensity physical activity, and MVPA were determined to be ≤ 288 counts, 289–766 counts, and ≥ 767 counts per five seconds based on vector magnitude, respectively [26]. The sum of light intensity physical activity and MVPA was defined as total physical activity. Sleep duration was estimated using ActiLife software v6.13.4 in 60-s epochs. Bedtime and wakeup time were identified using the automated Sadeh et al. algorithm [27], and sleep duration was detected using the Tudor-Locke algorithm [28], with visual inspection as a supplement.

Parents reported sedentary screen time of preschoolers using questions modified from the Children’s Leisure Activities Study Survey questionnaire-Chinese version, including the time spent on TV/DVDs, video games/computers, tablets/mobile phones [29].

### Covariates

A set of covariates was reported by parents, including preschoolers’ characteristics (age, sex, height, weight, number of siblings, eating habits), parents’ characteristics (age, sex, height, weight, education level), family income, family size, family structure, and type of residence. Body mass index was determined as weight (kg)/height (m^2^).

### Feasibility and acceptability

Feasibility and acceptability were assessed via retention rates, intervention fidelity, satisfaction, and usefulness of the intervention. More details of feasibility and acceptability evaluation are described elsewhere [18].

### Statistical analysis

Variables of interest were expressed as mean ± standard deviation (SD; continuous data) or number and percentage of preschoolers/parents (categorical data). Intention-to-treat analyses were adopted. Differences in demographics and movement behaviours across three groups at baseline were assessed using analysis of variance or Chi-Square tests. To examine the effects of the intervention (integrated approach vs. control group, dyadic approach vs. control group, dyadic approach vs. integrated approach) on outcomes (children’s overall movement behaviours, movement behaviour composition, total physical activity, MVPA, sedentary time, screen time, sleep duration), generalised estimating equations were applied using group as main effect, adjusting for covariates (preschoolers’ characteristics [age, sex, number of siblings, eating habits], parents’ characteristics [age, sex, body mass index, education level], family income, family size, family structure, and type of residence) and baseline outcome. Clustering was not included as a covariate in the analysis as a common practice for family-based interventions [30, 31]. All available data were used in generalised estimating equations without imputing missing values, as generalised estimating equation applies a natural and appropriate way to accommodate missing data [32]. Besides, per-protocol analysis (participants with complete data at all assessments) was conducted to compare primary outcomes between groups. Analyses were performed using SPSS 27.0. The significance level was set at 0.05. The effect of intervention on outcomes was presented as effect size (Cohen’s *d*), which was categorised as small, medium, and large, corresponding to 0.2, 0.5, and 0.8, respectively [33].

## Results

### Baseline characteristics

**Fig. 1:**
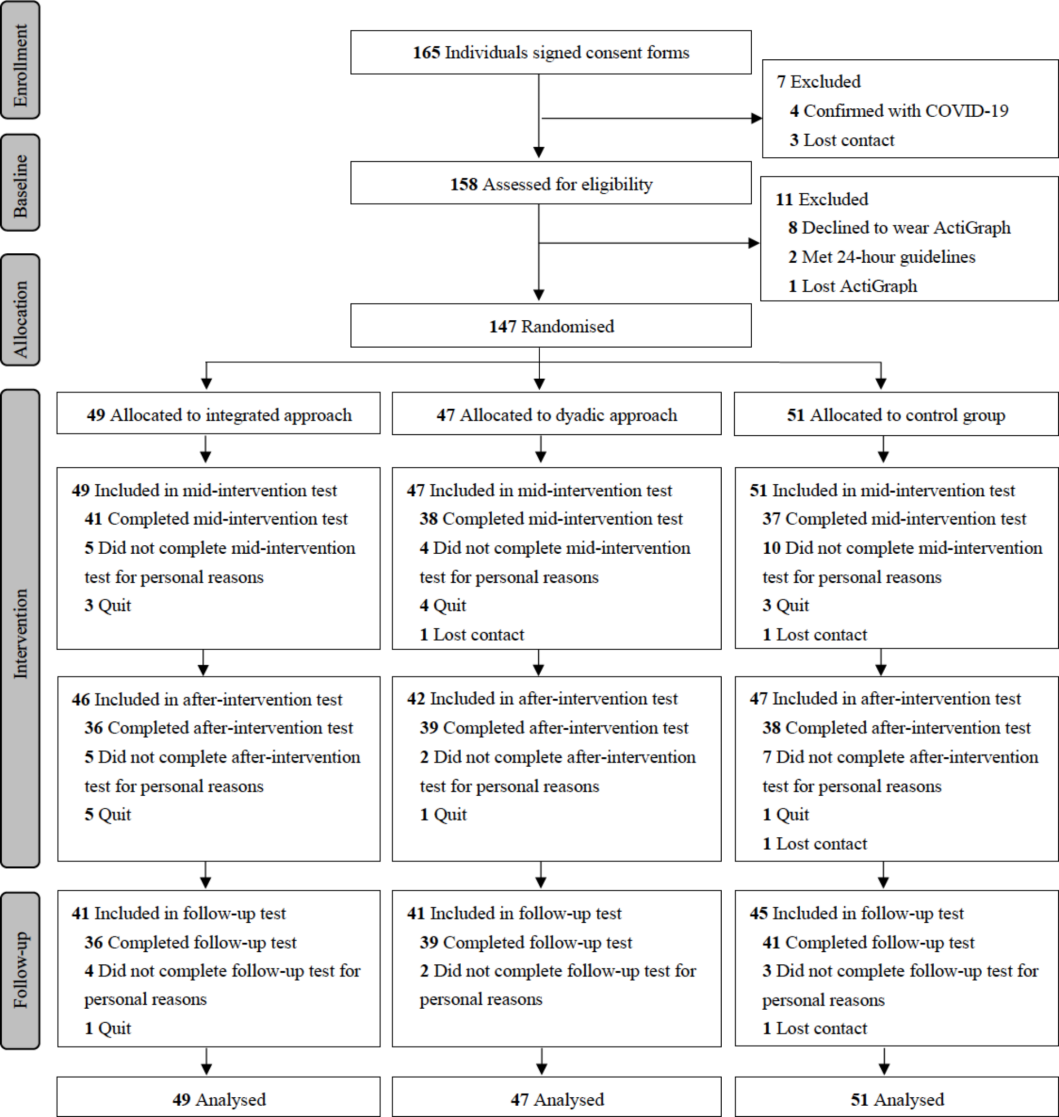
Flow chart of the study

A total of 165 parent-child pairs consented, and 158 of them were assessed for eligibility (Fig. 1). Eventually, 147 eligible parent-child pairs (preschoolers: 4.8 ± 0.9 years old, 56.5% boys; parents: 37.3 ± 5.3 years old, 15% fathers) were randomly assigned to the integrated approach (*n* = 49), dyadic approach (*n* = 47), or wait-list control group (*n* = 51). Descriptive statistics of the demographics at baseline are provided in Table 1, and there were no significant differences in demographic factors across the three groups except for the age of children and proportion of participating fathers. No demographic differences were found between those who completed all the tests across four time points and those who were lost to follow up.

**Table 1:** Summary of baseline characteristics of participants

	Integrated (*n* = 49)	Dyadic (*n* = 47)	Control (*n* = 51)
**Children’s characteristics**			
Age, years Mean ± SD	5.0 ± 1.0	4.9 ± 0.9	4.6 ± 0.9
Boys, No. (%)	29 (59.2%)	23 (48.9%)	31 (60.8%)
Body mass index	16.1 ± 3.3	15.4 ± 2.2	15.6 ± 2.6
Number of siblings	0.7 ± 0.8	0.8 ± 0.6	0.9 ± 0.6
Eating habits, No. (%)			
*Breakfast*			
≤ 7 days/week	7 (14.3%)	5 (10.6%)	7 (13.7%)
7 days/week	42 (85.7%)	42 (89.4%)	44 (86.3%)
*Fruit*			
< 1 serving/day	9 (18.4%)	9 (19.1%)	19 (37.3%)
1–2 servings/day	32 (65.3%)	32 (68.1%)	22 (43.1%)
≥ 3 servings/day	8 (16.3%)	6 (12.8%)	10 (19.6%)
*Vegetables*			
< 1 serving/day	18 (36.7%)	9 (19.1%)	19 (37.3%)
1–2 servings/day	23 (46.9%)	28 (59.6%)	16 (31.4%)
≥ 3 servings/day	8 (16.3%)	10 (21.3%)	16 (31.4%)
*Dairy products*			
< 1 serving/day	11 (22.4%)	12 (25.5%)	12 (23.5%)
1–2 servings/day	30 (61.2%)	27 (57.4%)	26 (51.0%)
≥ 3 servings/day	8 (16.3%)	8 (17.0%)	13 (25.5%)
*High-energy-density foods*			
< 1 serving/day	23 (46.9%)	22 (46.8%)	25 (49.0%)
1–2 servings/day	19 (38.8%)	20 (42.6%)	15 (29.4%)
≥ 3 servings/day	7 (14.3%)	5 (10.6%)	11 (21.6%)
Wear time of accelerometer (min)	1242 ± 203	1235 ± 235	1226 ± 226
**Parents’ characteristics**			
Age, years Mean ± SD	36.7 ± 4.8	37.2 ± 5.0	37.8 ± 6.1
Fathers, No. (%)	11 (22.4%)	2 (4.3%)	9 (17.6%)
Body mass index	22.1 ± 3.2	21.6 ± 3.6	21.5 ± 3.2
*Parents’ education level*,* No. (%)*			
Below degree	26 (53.1%)	20 (42.6%)	22 (43.1%)
Degree or higher	23 (46.9%)	27 (57.4%)	29 (56.9%)
*Family income*,* No. (%)*			
Low income	18 (36.7%)	10 (21.3%)	16 (32.0%)
Medium income	8 (16.3%)	11 (23.4%)	15 (30.0%)
High income	23 (46.9%)	26 (55.3%)	19 (38.0%)
Family size	4.3 ± 0.9	4.5 ± 1.1	4.5 ± 1.1
*Family structure*,* No. (%)*			
Nuclear family	37 (75.5%)	35 (74.5%)	42 (82.4%)
Extended family	10 (20.4%)	10 (21.3%)	9 (17.6%)
Others	2 (4.1%)	2 (4.3%)	0 (0%)
*Type of residence*,* No. (%)*			
Public rental housing	11 (22.4%)	13 (27.7%)	16 (31.4%)
Home ownership housing	6 (12.2%)	7 (14.9%)	7 (13.7%)
Private housing	25 (51.0%)	22 (46.8%)	22 (43.1%)
Others	7 (14.3%)	5 (10.6%)	6 (11.8%)

Descriptive statistics for all outcomes for each group at each time point are presented in Table 2. There were no significant differences in movement behaviours across the three groups at baseline. Compared with baseline, the preschoolers in all of the groups decreased in MVPA at postintervention and follow-up; the preschoolers in both intervention groups had lower ASI and screen time at postintervention and follow-up.

**Table 2:** Summary of descriptive data for each outcome variable (*n* = 147)

	Mean ± SD				Mean difference (95% CI)			
	Baseline	Postintervention	Follow-up		Postintervention vs. Baseline	*P* value	Follow-up vs. Baseline	*P* value
**Activity sleep index** ^a^								
Integrated	38.8 ± 4.4	36.8 ± 5.7	36.6 ± 5.3		–2.1 (–4.1, −0.0)	0.049	–2.2 (–4.4, −0.1)	0.042
Dyadic	39.7 ± 4.3	36.8 ± 5.6	36.5 ± 5.2		–2.9 (–4.7, −1.2)	0.001	–3.2 (–5.2, −1.2)	0.002
Control	37.7 ± 5.8	36.4 ± 5.1	35.2 ± 5.7		–1.3 (–2.9, 0.3)	0.111	–2.6 (–4.9, −0.2)	0.035
**Isometric log-ratio-Physical activity**								
Integrated	–0.37 ± 0.32	–0.47 ± 0.33	–0.48 ± 0.21		–0.09 (–0.24, 0.05)	0.214	–0.11 (–0.22, 0.00)	0.055
Dyadic	–0.33 ± 0.26	–0.52 ± 0.43	–0.54 ± 0.36		–0.18 (–0.32, −0.05)	0.007	–0.21 (–0.33, −0.08)	0.001
Control	–0.36 ± 0.29	–0.59 ± 0.45	–0.71 ± 0.50		–0.23 (–0.40, −0.07)	0.004	–0.35 (–0.54, −0.17)	< 0.001
**Isometric log-ratio-Sedentary behaviour**								
Integrated	–0.05 ± 0.34	–0.04 ± 0.37	0.01 ± 0.31		0.01 (–0.14, 0.17)	0.869	0.06 (–0.07, 0.18)	0.401
Dyadic	–0.03 ± 0.27	–0.04 ± 0.33	0.02 ± 0.24		–0.02 (–0.13, 0.10)	0.803	0.05 (–0.06, 0.15)	0.363
Control	–0.05 ± 0.27	0.10 ± 0.26	0.23 ± 0.31		0.15 (0.02, 0.27)	0.023	0.28 (0.15, 0.41)	< 0.001
**Isometric log-ratio-Sleep**								
Integrated	0.42 ± 0.30	0.50 ± 0.35	0.48 ± 0.29		0.08 (–0.06, 0.22)	0.247	0.06 (–0.06, 0.17)	0.357
Dyadic	0.36 ± 0.31	0.56 ± 0.39	0.52 ± 0.35		0.20 (0.06, 0.34)	0.005	0.16 (0.03, 0.29)	0.017
Control	0.41 ± 0.27	0.49 ± 0.37	0.48 ± 0.43		0.09 (–0.01, 0.19)	0.089	0.07 (–0.09, 0.24)	0.380
**Moderate-to-vigorous intensity physical activity**,** min/day**								
Integrated	95 ± 33	82 ± 31	77 ± 27		–13 (–26, −1)	0.034	–19 (–30, −7)	0.001
Dyadic	98 ± 30	82 ± 39	75 ± 34		–16 (–27, −5)	0.004	–22 (–33, −11)	< 0.001
Control	94 ± 33	76 ± 39	68 ± 35		–18 (–29, −7)	0.001	–26 (–38, −13)	< 0.001
**Total physical activity, min/day**								
Integrated	312 ± 87	289 ± 88	284 ± 58		–22 (–60, 15)	0.241	–27 (–57, 2)	0.072
Dyadic	331 ± 81	276 ± 108	268 ± 92		–55 (–87, −23)	< 0.001	–63 (–96, −30)	< 0.001
Control	318 ± 85	264 ± 106	245 ± 107		–54 (–86, −23)	< 0.001	–73 (–112, −34)	< 0.001
**Sedentary behaviour**,** min/day**								
Integrated	405 ± 108	408 ± 115	435 ± 119		3.2 (–45, 52)	0.898	30 (–17, 76)	0.207
Dyadic	425 ± 110	387 ± 112	405 ± 103		–38 (–81, 6)	0.090	–20 (–64, 24)	0.380
Control	408 ± 111	427 ± 99	473 ± 128		19 (–23, 60)	0.377	64 (11, 118)	0.018
**Screen time**,** min/day**								
Integrated	151 ± 109	100 ± 64	112 ± 91		–51 (–72, −29)	< 0.001	–39 (–66, −13)	0.004
Dyadic	121 ± 119	80 ± 65	75 ± 60		–41 (–64, −18)	< 0.001	–47 (–74, −19)	< 0.001
Control	130 ± 129	137 ± 102	138 ± 102		6 (–22, 35)	0.666	7 (–23, 38)	0.635
**Sleep duration**,** min/day**								
Integrated	572 ± 83	607 ± 106	617 ± 104		35 (–3, 73)	0.070	45 (4, 85)	0.029
Dyadic	570 ± 93	607 ± 103	593 ± 88		37 (–0, 74)	0.051	23 (–6, 52)	0.116
Control	570 ± 59	580 ± 98	564 ± 95		9 (–22, 41)	0.553	–6 (–43, 31)	0.743

^a^ The total index ranges from 0 to 60, and a higher index indicates healthier movement behaviours

### Effect on primary outcomes

Differences in the primary outcomes across the groups are presented in Table 3. At postintervention, no significant between-group differences were found, with consistent findings observed across both intention-to-treat and per-protocol analyses. At follow-up, the integrated approach group demonstrated higher ASI (integrated vs. control: 3.4; 95% confidence interval [CI]: 0.1 to 6.8; *d* = 0.26). Both intervention groups showed significantly higher ilr-physical activity (integrated vs. control: 0.33; 95% CI: 0.09 to 0.56; *d* = 0,59; dyadic vs. control: 0.25; 95% CI: 0.04 to 0.45; *d* = 0.40), and the dyadic approach reported lower ilr-sedentary behaviour at follow-up (dyadic vs. control: − 0.21; 95% CI: − 0.37 to − 0.05; *d* = 0.76), than control group. No significant differences were found for ilr-sleep. Per-protocol analyses, which included participants with complete data, yielded consistent results, except for the differences between the integrated and control group in ASI and ilr-sleep. Specifically, no significant difference was found in ASI (integrated vs. control: 2.42; 95% CI: − 1.50, 6.34; *d* = 0.36); while the integrated group showed lower ilr-sleep at follow-up (integrated vs. control: − 0.27; 95% CI: − 0.51, − 0.03; *d* = 0.11). Detailed results of the per-protocol analyses can be found in Supplementary Table 2.

**Table 3:** Generalised estimating equations model estimates of the differences in movement behaviours between groups (*n* = 147)

	Postintervention			Follow-up		
	Mean difference (95% CI)	*P* value	Effect size	Mean difference (95% CI)	*P* value	Effect size
**Activity sleep index** ^a^						
Integrated vs. Control	1.3 (–1.9, 4.6)	0.413	0.07	3.4 (0.1, 6.8)	0.046	0.26
Dyadic vs. Control	0.2 (–1.9, 2.4)	0.828	0.07	1.5 (–1.2, 4.2)	0.276	0.25
Dyadic vs. Integrated	–1.1 (–3.7, 1.5)	0.411	0.00	–1.9 (–5.0, 1.2)	0.227	0.02
**Isometric log-ratio-Physical activity**						
Integrated vs. Control	0.08 (–0.11, 0.27)	0.392	0.31	0.33 (0.09, 0.56)	0.007	0.59
Dyadic vs. Control	0.07 (–0.11, 0.25)	0.434	0.17	0.25 (0.04, 0.45)	0.018	0.40
Dyadic vs. Integrated	–0.01 (–0.19, 0.16)	0.906	0.13	–0.08 (–0.25, 0.08)	0.332	0.19
**Isometric log-ratio-Sedentary behaviour**						
Integrated vs. Control	–0.18 (–0.37, 0.00)	0.053	0.42	–0.15 (–0.32, 0.02)	0.081	0.72
Dyadic vs. Control	–0.07 (–0.21, 0.08)	0.360	0.46	–0.21 (–0.37, −0.05)	0.013	0.76
Dyadic vs. Integrated	0.12 (–0.07, 0.30)	0.218	0.02	–0.06 (–0.20, 0.08)	0.397	0.05
**Isometric log-ratio-Sleep**						
Integrated vs. Control	0.10 (–0.06, 0.27)	0.217	0.03	–0.16 (–0.38, 0.07)	0.166	0.00
Dyadic vs. Control	–0.00 (–0.13, 0.13)	0.979	0.17	–0.03 (–0.23, 0.17)	0.789	0.11
Dyadic vs. Integrated	–0.11 (–0.26, 0.05)	0.179	0.14	0.13 (–0.06, 0.32)	0.168	0.13
**Moderate-to-vigorous intensity physical activity**,** min/day**						
Integrated vs. Control	9 (–6, 24)	0.228	0.17	22 (4, 40)	0.015	0.27
Dyadic vs. Control	5 (–9, 18)	0.497	0.15	8 (–7, 23)	0.306	0.21
Dyadic vs. Integrated	–4 (–19, 10)	0.539	0.00	–14 (–30, 2)	0.087	0.03
**Total physical activity**,** min/day**						
Integrated vs. Control	18 (–28, 63)	0.447	0.26	72 (17, 127)	0.010	0.46
Dyadic vs. Control	8 (–31, 48)	0.673	0.12	24 (–27, 74)	0.355	0.23
Dyadic vs. Integrated	–9 (–50, 31)	0.655	0.13	–48 (–97, 0)	0.052	0.21
**Sedentary behaviour**,** min/day**						
Integrated vs. Control	–52 (–120, 16)	0.134	0.18	8 (–69, 85)	0.838	0.31
Dyadic vs. Control	–24 (–75, 27)	0.361	0.37	–67 (–143, 9)	0.086	0.59
Dyadic vs. Integrated	28 (–34, 90)	0.377	0.18	–75 (–137, −13)	0.018	0.27
**Screen time**,** min/day**						
Integrated vs. Control	–33 (–64, −1)	0.044	0.42	–30 (–59, −1)	0.041	0.27
Dyadic vs. Control	–31 (–54, −8)	0.010	0.66	–42 (–67, −17)	< 0.001	0.75
Dyadic vs. Integrated	2 (–22, 26)	0.897	0.31	–12 (–33, 10)	0.281	0.49
**Sleep duration**,** min/day**						
Integrated vs. Control	38 (–11, 87)	0.127	0.27	15 (–43, 74)	0.606	0.53
Dyadic vs. Control	–12 (–50, 26)	0.532	0.27	–13 (–63, 38)	0.622	0.31
Dyadic vs. Integrated	–50 (–100, −0)	0.050	0.01	–28 (–85, 29)	0.332	0.25

^a^ The total index ranges from 0 to 60, and a higher index indicates healthier movement behaviours

All models were adjusted for preschoolers’ characteristics (age, sex, number of siblings, eating habits), parents’ characteristics (age, sex, body mass index, education level), family income, family size, family structure, type of residence, and baseline outcome

### Effect on secondary outcomes

At postintervention, the integrated and dyadic approach groups had lower screen time than the control group (integrated vs. control: − 33 min/day; 95% CI: − 64 to − 1; *d* = 0.42; dyadic vs. control: − 31 min/day; 95% CI: − 54 to − 8; *d* = 0.66) (Table 3). Compared with the dyadic approach, the integrated approach showed a longer sleep duration at postintervention. No significant differences were found for the other secondary outcomes at postintervention. At follow-up, children’s MVPA and total physical activity were higher in the integrated approach group than those in control group. Both intervention groups had reduced screen time at follow-up. Compared with the integrated approach group, the dyadic approach group showed lower sedentary behaviour at follow-up. As for sleep duration, the improvement in the integrated approach group was not robust enough to yield a significant difference relative to either the dyadic approach group or the control group at follow-up.

### Feasibility and acceptability

Of the 147 parent-child pairs who were randomised, 18 (12.2%) withdrew for personal reasons (e.g., dropped out of school, transferred to another school, diagnosed with COVID-19, refused to wear the ActiGraph), and four (2.7%) lost contact. Finally, 116 (78.9%) parent-child pairs completed the follow-up test, while nine (6.1%) parent-child pairs did not complete the test for personal reasons (e.g., diagnosed with COVID-19, busy schedule).

Educational materials, reports, interactive questionnaires, and reminders were delivered to all of the participating families in a timely manner. In the integrated approach group, 18 (36.7%), 17 (34.7%), and 15 (30.6%) parents attended the first, second, and third workshops, respectively, and recorded videos of each workshop were sent to the parents who did not attend. In the dyadic approach group, 18 parents participated in each of the three online workshops, and the remaining parents received recorded videos of the workshops.

More than three fifths of the parents rated themselves as ‘very satisfied’ or ‘satisfied’ with the physical activity component (integrated approach: 67%; dyadic approach: 66%), the sedentary behaviour component (integrated approach: 67%; dyadic approach: 82%), and the sleep component (integrated approach: 75%). All intervention approaches were rated as a moderate level of satisfaction. Specifically, of those receiving the intervention, 63% found the educational materials to be ‘useful’ or ‘very useful’, 55% indicated that the workshops were ‘useful’ or ‘very useful’, 74% found the reports to be ‘useful’ or ‘very useful’, and 56% found the interactive questionnaires ‘useful’ or ‘very useful’.

## Discussion

To the best of our knowledge, this study is the first attempt to explore the effectiveness of an integrated movement behaviour intervention (targeting physical activity, sedentary behaviour, and sleep simultaneously) on promoting overall 24-hour movement behaviours (based on ASI and compositional data) and to compare the effectiveness of the integrated and dyadic approaches among preschoolers in Hong Kong. We found that overall movement behaviours of preschoolers changed unfavourably over the 24-week period. However, these changes were mitigated at follow up in the integrated intervention group, which partially supports our hypothesis that integrated approach is effective in improving young children’s overall movement behaviours. Both interventions mitigated the decline of the composition of time spent in physical activity and reduced screen time, while only the dyadic intervention approach further mitigated the increase of sedentary behaviour subcomponent. No significant changes were found in sleep in both intervention groups. The high retention rate and positive feedback support the feasibility and acceptability of a 24-hour movement behaviour intervention among parents with a preschool-aged child.

A less favourable overall behaviour profile (ASI) across time mainly driven by physical activity dimensions was observed in all groups, indicating that preschoolers’ overall movement behaviours became unhealthier over the 24-week period. High baseline physical activity levels in the sample in this study possibly leave little room for improvement in terms of physical activity. Specifically, the present study observed higher total physical activity levels at baseline compared to two previous studies [34, 35]. However, it is important to note that the observed discrepancy may be partly attributable to differences in the wear location of ActiGraph accelerometers (wrist vs. waist), participant characteristics (age, sex distribution), and data collection period (May to July 2022 vs. June 2020) between the present study and that by Ng et al. [34]. Additionally, the other study relied on parent-reported questionnaires to measure physical activity [35]. Nevertheless, both of the previous studies observed levels of MVPA similar to ours [34, 35]. Another possible explanation involves age-related decline: a systematic review of 52 studies involving 3- to 18-year-old participants summarised device-based evidence of longitudinal changes in physical activity, and reported a significant decline in MVPA over time [36]. This evidence sounds an alarm, reminding that physical activity may decline significantly without intervention in the early years. However, the effectiveness of the intervention needs to be further explored among preschoolers who are less physically active.

Preschoolers in the integrated approach had a higher ASI at follow-up compared with the control group with a small effect size, partially supports the hypothesis. ASI provides a total score for various dimensions of movement behaviours, with a higher score indicating better overall movement behaviours [22]. This indicator therefore reflects the overall pattern of 24-hour movement behaviours (total physical activity, MVPA, screen time, and sleep duration and timing) and provides insight on how various behaviours change simultaneously. However, most of the existing evidence on the effectiveness of movement behaviour interventions has been specific to individual behaviours [11–13], and no study has simultaneously targeted all three 24-hour movement behaviours using integrated strategies for children in the early years. Among adolescents, an intervention adopting a quasi-experimental design aimed at improving all three 24-hour movement behaviours, diet, and substance use simultaneously showed an improvement in meeting the 24-hour movement behaviour guidelines after one academic year [8]. In the present study, an unfavourable change to a lesser extent on ASI was only observed in the integrated approach, further highlighting the advantage of targeting all three 24-hour movement behaviours in an intervention. Furthermore, preschoolers in the integrated approach managed to maintain a relatively higher ASI at follow-up, indicating a sustained beneficial effect on movement behaviours. This may have contributed to the parent-child pairs’ self-motivated, continued practice of intervention content [37]. However, these findings should be interpreted with caution and require future studies that monitor parental practice during the follow-up period.

Compared with the control group, both intervention approaches were effective in mitigating the reduction of the composition of time spent in physical activity at follow-up (small to moderate effect size), supporting our hypotheses. Previous studies have used compositional data analysis when examining association between 24-hour movement behaviours and health outcomes among preschool-aged children, but most of them have applied cross-sectional design and measured sleep duration subjectively [38, 39]. There have been a limited number of studies examining the effect of interventions on the composition of movement behaviours; a behaviour change intervention on children aged 8–9 years old revealed no significant changes in the distribution of movement behaviours after a 18-month school- and home-based intervention [40]. Therefore, it is difficult to make direct comparisons with the aforementioned study due to the different target age groups.

Another noticeable finding of the intervention was the reduction in screen time, with small (integrated approach) or medium to large (dyadic approach) effect sizes. Among all the outcomes, screen time was the only outcome that demonstrated significant decrease at the completion of the intervention (12 weeks). Compared with the control group, preschoolers in both intervention groups spent at least 30 min/day less screen time than the control group at both postintervention and follow-up. These findings supported our hypothesis and previous studies [41, 42]. A systematic review of interventions aiming at reducing screen time showed a pooled reduction in screen time of three to four hours per week among preschool-aged children [41]. Furthermore, another systematic review found that with the intervention components planning, goals, and feedback, interventions lasting less than or equal to 12 weeks were effective in reducing screen time [42]. Given the negative effects on health of high amounts of screen time [3] and the significant effectiveness of the present intervention on reducing screen time, it seems important to complete interventions to manage screen time in the early years of childhood.

As for sleep duration, although the integrated approach did not yield a statistically significant improvement compared with control group, it did demonstrate longer sleep duration at both postintervention and follow-up, with small-to-medium effect sizes. In addition, compared with baseline, the integrated approach showed an increase in sleep duration at both postintervention (35 min/day) and follow-up (45 min/day), and the improvement was significant at follow-up, partially supporting our hypothesis. A systematic review of sleep interventions showed a small but significant improvement in sleep duration in a 24-hour cycle in the early years, but most of the evidence was based on sleep diaries [13]. Although there has been no consensus on the minimum intervention duration to achieve considerable improvement in sleep among children or adults [13], it is plausible that a longer time is needed to change sleep patterns. Furthermore, compared with the dyadic approach, the integrated approach had 50 min a day more sleep duration at postintervention, but such difference was not significant at follow-up. Above all, there was insufficient evidence to conclude the effectiveness of the present intervention on sleep, and further interventions with a longer duration and targeting improving sleep duration are warranted.

Our hypothesis that the integrated approach would yield greater effectiveness in positively changing movement behaviours than dyadic approach was generally not supported by the findings except for the absolute durations of sedentary behaviour at follow-up and sleep after the intervention. The dyadic approach showed a greater reduction in sedentary behaviour at follow-up (albeit with a small effect size), with a shorter sleep duration at postintervention, compared with the integrated approach. This might be explained by the heavy workload for families in the integrated approach, as different resources and strategies were provided to improve each behaviour, which may lead to confusion in the prioritisation of interventions and preschoolers may focus on one or two behaviours only. Furthermore, targeting physical activity and sedentary behaviour only may not necessarily lead to improved sleep [43]. In view of the above, it is important to recognise the challenges when handling the interplay and trade-offs between these behaviours in multi-behaviour interventions. Future interventions should be developed with a tailored approach to suit individuals’ needs.

Our findings suggest good feasibility of the intervention as demonstrated by the acceptable retention rate. In addition to the common reasons for withdrawing from interventions, the pandemic posed another challenge, as some of the parent-child pairs could not complete the assessments because they had been diagnosed with COVID-19. The fidelity and adherence were established with timely delivery of education materials, workshops, reports, and interactive questionnaires. However, approximately half of the parents in both intervention groups did not attend online interactive workshops due to busy schedules or time conflicts, instead receiving recorded videos of the workshops and messages as a substitute. Regarding the interactive questionnaires, 43% (integrated approach) and 47% (dyadic approach) of parents have utilised them for self-monitoring and planning. Furthermore, the favourable responses from the parents to the intervention evaluation conducted upon the completion of the intervention showed that this intervention was acceptable for the preschoolers. Positive feedback on the usefulness of individual reports on the children’s current movement behaviours indicated the importance of timely feedback, which has been suggested by a previous systematic review [44]. Overall, this online intervention was considered feasible and acceptable by the parents, indicating that the adoption of a parent-focused intervention targeting multiple behaviours has the potential to be promoted among preschoolers.

The strengths of this study include targeting all three 24-hour movement behaviours, utilising the Internet-based delivery, adopting a theoretical framework, using device-based measurement of children’s movement behaviours, and applying a randomised controlled trial design. However, there are some limitations. Although expected [21], one fifth of the parent-child pairs did not complete the follow-up test due to voluntary withdrawal, losing contact, or other reasons (e.g., contracting COVID-19). To avoid biases that may arise from attrition, the intention-to-treat principle was applied in this study. Secondly, to include as many participants as possible in the analysis, this study required at least one day of valid ActiGraph data. Although this criterion has been used previously [24], data based on more robust inclusion criteria would better reflect preschoolers’ movement behaviour patterns. Nonetheless, the preschoolers in the present study had 6.1 valid days of data on average, suggesting that the bias is minor. Thirdly, the nap duration was not captured in this study, which may have led to a potential impact on the findings related to overall sleep. Lastly, the generalisation of the findings is limited because of the characteristics of the sample (a high level of baseline physical activity) and the data collection period (transitioning out of the COVID-19 pandemic). The pandemic-induced precautions may have affected the baseline levels of children’s movement behaviours and limited options for improving children’s movement behaviours. Future studies should incorporate face-to-face elements, such as interactive workshops and individual consultations, to complement the current online intervention. Blending digital and in-person delivery modes may enhance the intervention’s reach and effectiveness, though further investigation is needed to determine the optimal combination of these components.

Considering the favourable effects on overall movement behaviours observed at follow-up, the adoption of an integrated intervention targeting all three 24-hour movement behaviours has the potential to promote a holistic, healthy lifestyle among preschoolers. Given the similar demographic characteristics (e.g., age, gender distribution, body mass index) of the sample in this study compared to other studies targeting preschoolers in Hong Kong and mainland China [7, 45], the findings have potential to be generalised to the broader preschool population in these regions. Future studies examining the effect of integrated interventions on health outcomes are needed to explore clinical implications.

## Conclusions

Both integrated and dyadic groups showed a decrease in screen time at postintervention, but did not show significant changes in other behaviours. Our intervention has demonstrated unfavourable changes in overall movement behaviours over 24 weeks among the preschoolers, but the changes were mitigated by the implementation of the integrated intervention. Both integrated and dyadic approaches alleviated the decline in the composition of time spent in physical activity and reduced screen time, and the dyadic approach further attenuated the increase in sedentary behaviour subcomponent among the preschoolers. The favourable effect on the overall movement behaviours of the integrated approach group supports the effectiveness of the 24-hour movement behaviour intervention among preschoolers. Further interventions with longer duration are warranted to explore effective strategies to induce changes in sleep.

## Electronic supplementary material

Below is the link to the electronic supplementary material.

Supplementary Material 1

Supplementary Material 2

Supplementary Material 3

## Data Availability

The datasets used and/or analysed during the current study are available from the corresponding author on reasonable request.

## Abbreviations

ASI: Activity Sleep Index
CI: Confidence interval
CONSORT: Consolidated Standards of Reporting Trials
COVID-19: Coronavirus Disease 2019
ilrs: isometric log-ratio coordinates
MVPA: Moderate-to-vigorous intensity physical activity
SD: Standard deviation
