# Supplementary material for: Effectiveness of a parent-focused intervention targeting 24-hour movement behaviours in preschool-aged children: a randomised controlled trial
Source: Int J Behav Nutr Phys Act. 2024 Sep 9;21:98. doi: 10.1186/s12966-024-01650-2 (PMC11385828; doi:10.1186/s12966-024-01650-2)
Supplement: Supplementary file 3 — Supplementary Material 3 [file 12966_2024_1650_MOESM3_ESM.docx]

Supplementary Table 1. Strategies and behaviour change techniques involved in the integrated approach

| Approach | Time | Strategies | Behaviour change technique (code) [17] | Contents |
| --- | --- | --- | --- | --- |
| Education materials | Week 1 & 2 | Attitude toward the behaviour, subjective norm, monitoring, goal setting | Information about health consequences (5.1)  Information of others’ approval (6.3)  Feedback on behaviour (2.2)  Discrepancy between current behaviour and goal (1.6)  Goal-setting (behaviour) (1.1) | 1. WHO 24-h movement guidelines for preschoolers  2. Benefits of having a healthy lifestyle (i.e., high physical activity, low sedentary behaviour, low screen time, sufficient sleep) for preschoolers  3. What important others think about the behaviour  4. Individual report on children’s current level of movement behaviours and the gap with the guidelines  5. Steps and strategies of goal-setting (e.g., SMART goals) |
|  | Week 3 & 4 | Planning, intention | Action planning (1.4)  Non-specific incentive (10.6) | 1. Examples of improving physical activity, reduce sedentary behaviour, and improve sleep  2. Examples of giving incentives for child when he/she make effort/progress in performing the behaviour |
|  | Week 5 & 6 | Perceived behavioural control, planning, intention | Problem-solving (1.2)  Action planning (1.4)  Non-specific incentive (10.6) | 1. Strategies to overcome barriers  2. Examples of improving physical activity, reduce sedentary behaviour, and improve sleep  3. Examples of giving incentives for child when he/she make effort/progress in performing the behaviour |
|  | Week 7 & 8 | Monitoring, perceived behavioural control, planning, habit development | Feedback on behaviour (2.2)  Problem-solving (1.2)  Action planning (1.4)  Habit formation (8.3) | 1. Individual report on children’s current level of movement behaviours and the gap with the guidelines  2. Strategies to overcome barriers  3. Examples of improving physical activity, reduce sedentary behaviour, and improve sleep  4. Examples of habits |
|  | Week 9 & 10 | Planning, habit development | Action planning (1.4)  Behavioural substitution (8.2)  Habit formation (8.3) | 1. Examples of improving physical activity, reduce sedentary behaviour, and improve sleep  2. Strategies for developing habits (substitution, repetition) |
|  | Week 11 & 12 | Monitoring, habit development | Feedback on behaviour (2.2)  Behavioural substitution (8.2)  Habit formation (8.3) | 1. Individual report on the change of children’s movement behaviours during the past 10 weeks (based on parent-reported interactive questionnaires)  2. Strategies for developing habits (substitution, repetition) |

Supplementary Table 1. *(Continued)*

| Workshops | Week 1 | Subjective norm, attitude toward the behaviour, goal setting | Information of others’ approval (6.3)  Information about health consequences (5.1)  Goal-setting (behaviour) (1.1) | 1. Introduction of the program  2. WHO 24-h movement guidelines for preschoolers  3. Benefits of having a healthy lifestyle (i.e., high PA, low SB, low screen time, sufficient sleep) for preschoolers  4. Steps and strategies of goal setting |
| --- | --- | --- | --- | --- |
|  | Week 3 | Perceived behavioural control, intention | Problem-solving (1.2)  Verbal persuasion about capability (15.1) | 1. Solve problems that families have faced using materials  2. Provide strategies to parental perceived barriers  3. Sharing experiences  4. Encouraging families to comply with the intervention |
|  | Week 6 | Perceived behavioural control, intention, habit development | Problem-solving (1.2)  Verbal persuasion about capability (15.1)  Habit formation (8.3) | 1. Provide strategies to parental perceived barriers  2. Sharing experiences  3. Encouraging families to comply with the intervention  4. Example of habits  5. Strategies for developing habits |
| Interactive questionnaires | Week 2, 4, 6, 8, & 10 | Goal setting, monitoring | Review behaviour goals (1.5)  Self-monitoring of behaviour (2.3) | 1. Movement behaviours over the past two weeks (duration, whether achieve the goal or not)  2. Bi-weekly goals for each movement behaviour  3. Reminders of materials use |

Abbreviations: BCT, behaviour change technique; PA, physical activity; SB, sedentary behaviour; SMART, specific, measurable, achievable, realistic, timely; WHO, World Health Organisation.

Source: Feng J, Huang WY, Sit CH-P. Effectiveness of a parent-focused intervention targeting 24-h movement behaviors in preschool-aged children: Study protocol for a randomized controlled trial. Front Public Health. 2022;10:870281. Copyright © 2022 Feng, Huang and Sit. Reprinted with permission.

Supplementary Table 2

Generalised estimating equations model estimates of the differences in primary outcomes (n = 105).

|  | Postintervention | |  | Follow-up | |  |
| --- | --- | --- | --- | --- | --- | --- |
|  | Mean difference (95% CI) | P value | Effect size | Mean difference (95% CI) | P value | Effect size |
| **Activity sleep index ^a^** |  |  |  |  |  |  |
| Integrated vs. Control | 0.67 (–2.48, 3.81) | 0.678 | 0.38 | 2.42 (–1.50, 6.34) | 0.226 | 0.36 |
| Dyadic vs. Control | 1.04 (–1.13, 3.21) | 0.349 | 0.24 | 1.16 (–1.83, 4.15) | 0.448 | 0.26 |
| Dyadic vs. Integrated | 0.37 (–2.47, 3.22) | 0.797 | 0.15 | –1.26 (–5.01, 2.48) | 0.508 | 0.10 |
| **Isometric log-ratio-Physical activity** |  |  |  |  |  |  |
| Integrated vs. Control | 0.01 (–0.19, 0.20) | 0.953 | 0.24 | 0.29 (0.02, 0.56) | 0.037 | 0.51 |
| Dyadic vs. Control | 0.06 (–0.13, 0.26) | 0.509 | 0.28 | 0.27 (0.04, 0.51) | 0.022 | 0.45 |
| Dyadic vs. Integrated | 0.06 (–0.14, 0.26) | 0.565 | 0.03 | –0.01 (–0.20, 0.17) | 0.888 | 0.05 |
| **Isometric log-ratio-Sedentary behaviour** | |  |  |  |  |  |
| Integrated vs. Control | –0.15 (–0.35, 0.05) | 0.135 | 0.56 | –0.00 (–0.16, 0.16) | 0.991 | 0.52 |
| Dyadic vs. Control | –0.12 (–0.27, 0.04) | 0.147 | 0.66 | –0.13 (–0.25, –0.00) | 0.046 | 0.65 |
| Dyadic vs. Integrated | 0.03 (–0.17, 0.23) | 0.743 | 0.00 | –0.13 (–0.26, 0.00) | 0.054 | 0.08 |
| **Isometric log-ratio-Sleep** |  |  |  |  |  |  |
| Integrated vs. Control | 0.14 (–0.03, 0.31) | 0.096 | 0.19 | –0.27 (–0.51, –0.03) | 0.029 | 0.11 |
| Dyadic vs. Control | 0.04 (–0.08, 0.17) | 0.489 | 0.20 | –0.14 (–0.33, 0.06) | 0.181 | 0.03 |
| Dyadic vs. Integrated | –0.10 (–0.28, 0.08) | 0.290 | 0.03 | 0.13 (–0.07, 0.34) | 0.198 | 0.11 |

^a^ The total index ranges from 0 to 60, and a higher index indicates healthier movement behaviours.

All models were adjusted for preschoolers’ characteristics (age, sex, number of siblings, eating habits), parents’ characteristics (age, sex, body mass index, education level), family income, family size, family structure, type of residence, and baseline outcome.
